# AI-enabled passive digital phenotyping of experiential negative symptoms in schizophrenia-spectrum disorders: a narrative review on the path from sensing to clinical endpoints

**DOI:** 10.3389/fpsyt.2026.1898444

**Published:** 2026-09-17

**Authors:** Yanping Shu, Qing Li, Lifan Liang, Jiaoying Liu, Zuli Zheng, Tong Li, Sha Huang, Jiajing Chen, Juanrong Wen, Jiang Tan

**Affiliations:** 1 Department of Women and Child Psychiatry, The Second People’s Hospital of Guizhou Province, Guiyang, China; 2 The Second Clinical Medical College, Guizhou University of Traditional Chinese Medicine, Guiyang, China; 3 School of Digital Economy and Finance, Guizhou University of Commerce, Guiyang, China; 4 The First Clinical Medical College, Zunyi Medical University, Zunyi, China; 5 College of Medical Humanities, Guizhou Medical University, Guiyang, China; 6 School of Psychology, Guizhou Normal University, Guiyang, China

**Keywords:** artificial intelligence, asociality, avolition, digital endpoints, digital phenotyping, negative symptoms, passive sensing, schizophrenia-spectrum disorders

## Abstract

Negative symptoms, particularly avolition and asociality, rank among the most disabling and treatment-resistant features of schizophrenia-spectrum disorders. Clinician-rated scales such as the Clinical Assessment Interview for Negative Symptoms and the Brief Negative Symptom Scale have strengthened construct definition. However, they depend on infrequent interviews and retrospective recall, missing the moment-to-moment behavioral patterns that define these symptoms in everyday life. Passive digital phenotyping, through smartphones and wearable sensors, can continuously capture mobility, physical activity, sleep, social rhythms, and speech acoustics under real-world conditions. Whether artificial intelligence can convert these noisy, incomplete, and context-dependent data streams into clinically valid digital endpoints remains the central unresolved question. This narrative review synthesizes evidence on AI-enabled passive digital phenotyping of experiential negative symptoms in schizophrenia-spectrum disorders. We propose a construct-to-endpoint pipeline that links construct definition, multimodal representation learning, and fit-for-purpose clinical validation to interpretable digital markers and just-in-time adaptive interventions. Current evidence reveals consistent but modest associations between passive sensing modalities and negative symptoms. However, single-modality correlations do not constitute valid endpoints. A qualified digital endpoint must demonstrate analytical validity, clinical validity, longitudinal sensitivity, external transportability, and patient meaningfulness. Key challenges span data-level issues such as resolution mismatch and device heterogeneity, inferential risks including environmental confounding and algorithmic bias, and ethical-regulatory concerns involving privacy and model opacity. The field should now prioritize construct-anchored, context-aware, and interpretable digital endpoints that can serve as outcome measures in negative-symptom trials and support personalized longitudinal monitoring.

## 1 Introduction

Schizophrenia-spectrum disorders are heterogeneous conditions in which psychosis represents only one facet of the clinical burden (1). Among the persistent features that remain after acute psychotic episodes remit, experiential negative symptoms, particularly avolition and asociality, are primary determinants of functional disability, diminished social participation, and compromised quality of life (2, 3). These symptoms reflect impairments in motivation, goal-directed behavior, and social engagement, and they remain among the most treatment-resistant dimensions of the disorder.

Measurement limitations pose a scientific challenge, not just a technical problem. Modern negative-symptom scales, such as the Clinical Assessment Interview for Negative Symptoms (CAINS) and the Brief Negative Symptom Scale (BNSS), have advanced the field by differentiating experiential symptoms from expressive deficits (4–8). These instruments also minimize contamination from depression, medication side effects, and disorganization. These scales rely on infrequent interviews and retrospective recall, failing to capture the small, everyday decisions that characterize avolition and asociality, such as getting out of bed, answering a text, or starting a conversation. Mucci et al. observed that although remote digital phenotyping has entered the discourse on measurement, the field has yet to establish how digital measures should be anchored to negative-symptom constructs or effectively integrated into clinical trials (9).

Passive digital phenotyping can address this methodological gap. Smartphones and wearable devices enable continuous capture of movement, location, sleep, speech, screen interaction, and communication metadata at a temporal density unattainable through standard clinical assessment (10–13). However, these data points do not constitute symptoms in isolation. Reduced mobility can signal avolition, yet it can also alternatively stem from unemployment, neighborhood safety concerns, poverty, adverse weather, physical illness, medication effects, or personal choice. Similarly, limited social contact can indicate asociality, but often arises from social exclusion, hospitalization, cultural norms, or restricted opportunities for interaction. Consequently, the scientific objective extends beyond simply deploying sensors. The central aim is to determine whether passively measured behavior, when modeled alongside clinical and contextual information, can be transformed into an interpretable, patient-centered digital endpoint.

We synthesize evidence on whether artificial intelligence (AI)-enabled passive phenotyping can generate clinically valid endpoints for avolition and asociality in schizophrenia-spectrum disorders. Prior reviews have addressed feasibility, diagnostic classification, remote measurement challenges, and endpoint considerations for negative symptoms (14–20). Building on this foundation, we contribute an integrated construct-to-endpoint pipeline with explicit validation gates and an emphasis on context-aware, multimodal modeling for clinical trials and just-in-time adaptive interventions (JITAIs).

## 2 Methods

This review examines studies that link passive sensing, AI modeling, and negative symptom assessment in schizophrenia-spectrum disorders. We focused on work addressing clinical constructs (negative symptoms, avolition, asociality), sensing modalities (passive sensing, smartphones, wearables, GPS, accelerometry, speech), AI and machine-learning methods (representation learning, time-series modeling, multimodal learning), and validation frameworks relevant to digital endpoints (V3, FDA Digital Health Technologies guidance, TRIPOD+AI). We drew on empirical studies, systematic reviews, conceptual frameworks for digital phenotyping and negative-symptom assessment, and regulatory guidance for digital health technologies.

Evidence was synthesized based on a construct-to-endpoint logic. First, we examined the conceptualization and measurement of avolition and asociality. Second, we mapped passive sensing modalities to candidate behavioral expressions of these constructs. Third, we evaluated AI and machine-learning approaches capable of converting longitudinal, multimodal, and incomplete data streams into clinically interpretable markers. Fourth, we reviewed validation requirements for digital endpoints. Finally, we considered clinical translation, ethics, and future research priorities. This organizational framework was adopted because the field lacks sufficient maturity for a single pooled effect estimate, and because the primary challenge lies in translational validity rather than solely in empirical association.

## 3 Experiential negative symptoms as dynamic, context-dependent behavior

Negative symptoms are typically categorized into two dimensions: motivation and pleasure, and diminished expression (21, 22). The motivation and pleasure dimension includes avolition, asociality, and anhedonia. The diminished expression dimension includes blunted affect and alogia. The present review focuses on experiential symptoms because passive sensing is optimally suited to capture the behavioral manifestations of reduced initiation, sustained effort, approach, and everyday engagement. Avolition is defined as an impairment in the initiation and maintenance of goal-directed behavior. In daily life, this manifests as reduced activity diversity, restricted routines, fewer transitions between activities, prolonged sedentary periods, and decreased translation of intention into action. Asociality refers to a reduced desire for, engagement in, or pursuit of social interaction. In real-world data, this may appear as decreased time in social contexts, fewer social contacts, lower interaction diversity, reduced participation in social venues, and limited response to social opportunities. Anhedonia overlaps with both domains because reward anticipation, consummatory pleasure, and positive affect influence whether individuals seek, sustain, and repeat activities (23).

This dynamic perspective is critical because an average score on a negative-symptom scale conflates multiple time scales. An individual may exhibit a stable trait-level avolition burden alongside day-to-day fluctuations in energy and social approach, as well as momentary shifts tied to context, defeatist beliefs, environmental stress, or positive affect. EMA studies have demonstrated that negative symptoms vary across contexts; for example, Luther et al. showed that environmental context predicts state fluctuations in negative symptoms (24, 25). Furthermore, effort-based decision-making, reinforcement learning, and defeatist beliefs correlate with daily motivational states (26–30). These findings support the measurement of experiential negative symptoms as real-world trajectories rather than solely as retrospective summaries. However, the construct cannot be reduced to a single sensor stream. A restricted life-space radius may align with avolition, but it does not constitute avolition. Similarly, a low call count may be consistent with asociality, but it is not synonymous with the condition. Any mapping of constructs to signals must address three questions. First, which clinical construct is the signal intended to index? Second, what non-symptom factors could plausibly generate the same signal? Third, does the signal track outcomes that patients, clinicians, and trialists consider meaningful? Without this methodological rigor, passive sensing risks generating appealing but clinically superficial correlates. **Figure 1** illustrates this structured pathway from construct to endpoint, with validation gates at each stage (**Figure 1**).

**Figure 1:**
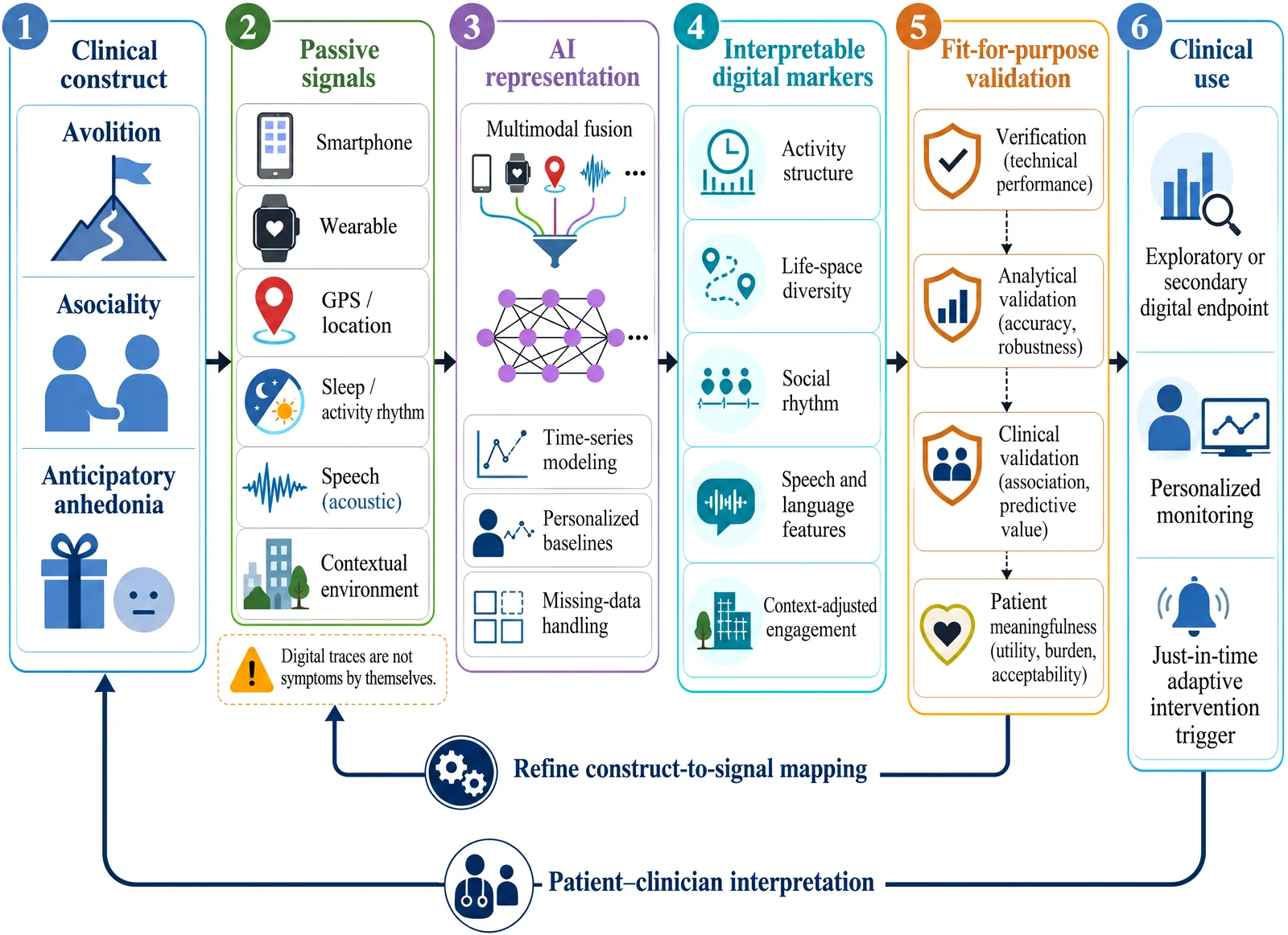
Construct-to-endpoint AI pipeline for experiential negative symptoms. This figure illustrates the pathway extending from the clinical construct through passive signals, AI representations, and interpretable digital markers to fit-for-purpose digital endpoints and JITAIs triggers. The pipeline incorporates validation gates for construct validity, technical and analytical validity, and clinical meaningfulness. Source: Created in Microsoft PowerPoint. Icons and illustrations were adapted from open-license resources, with modifications limited to size, color, layout, and style consistency.

Distinguishing diminished motivation from reduced opportunity remains central to valid negative-symptom assessment. Physical inactivity, for example, may stem from depression, sedation, medication side effects, or physical illness rather than avolition (see **Table 1**). Actigraphy and GPS therefore require clinical anchors such as CAINS, BNSS, and EMA to prevent conflating symptom severity with health status or environmental opportunity.

**Table 1:** Passive sensing modalities mapped to experiential negative symptoms.

Modality	Candidate digital phenotypes	Construct relevance	Key confounders and interpretation risks	Validation anchors
Geolocation and mobility	Time at home, number of locations, distance traveled, location entropy, life-space radius	Avolition, asociality, environmental exploration	Neighborhood safety, transportation, income, employment, disability, hospitalization, weather, culture	BNSS/CAINS motivation and pleasure (3–8, 22); geolocation-anchored negative symptoms and motivation/pleasure (36, 38, 39, 50, 91); EMA motivation and affective context (24–28, 37); functional capacity/functional outcome (2, 36, 37, 63); social functioning and social-network structure (42, 43, 63); patient-relevant life-space/activity outcomes (37, 66, 70, 71)
Wearable activity and sleep	Step count, sedentary bouts, activity fragmentation, circadian regularity, sleep timing and duration	Behavioral initiation, persistence, day structure, energy regulation	Sedation, extrapyramidal symptoms, medical illness, device nonwear, shift work, depression	Actigraphy/step-count convergence (31–33, 40, 70); negative-symptom ratings (2, 3, 40, 64); EMA-reported activity (37, 70, 71); treatment-response sensitivity (70–73)
Social sensing and communication metadata	Call or message frequency, response latency, interaction diversity, proximity to others, social-place visits	Asociality, social approach, social rhythm	Privacy risk, platform differences, social opportunity, cultural norms, stigma, limited data plans	Social network measures (42, 43, 63); EMA social context and loneliness (24, 25, 44, 71); patient-reported loneliness/social contact (44, 63); objectively sensed interaction and social functioning (43, 63)
Phone interaction and routine	Screen events, charging, app categories, unlock rhythm, daily regularity	Avolition, routine stability, behavioral inertia	Work demands, gaming, insomnia, phone sharing, digital literacy, socioeconomic status	Within-person baselines (51, 79, 81); data quality and missingness patterns (34, 59–61); EMA motivation/surveys (26, 27, 77, 78); clinical interviews and functioning ratings (36, 63, 64)
Speech, language, and conversational behavior	Pause duration, speech rate, prosody, lexical diversity, semantic coherence, turn-taking	Adjacent expressive symptoms, social communication, alogia, treatment monitoring	Language, dialect, recording context, medication, microphone quality, task effects	Standardized negative-symptom/expressive-domain anchors (3, 8, 45, 64); PANSS-based speech symptom ratings (46); longitudinal symptom change (45); standardized multisite speech/language collection and QA (47); cross-language validation/generalizability (48)
Environmental and contextual data	Weather, census context, neighborhood deprivation, ethnoracial congruence, activity-space resources	Context-sensitive engagement, social opportunity, structural constraints	Ecological fallacy, privacy, incomplete geocoding, confounding by residence	EMA state symptoms (24, 25); contextual exposures and geocoding (24, 25, 49); patient reports/contextual interpretation (24, 25, 49, 83, 86); multilevel or machine-learning models (50–53)

## 4 Multiple passive sensing modalities generate candidate phenotypes for experiential negative symptoms

Passive sensing in schizophrenia-spectrum disorders has progressed from feasibility studies to comprehensive longitudinal measurement. Research indicates that smartphones and wearable devices can capture rest-activity patterns, mobility, adherence, and symptom-relevant behavior in outpatient samples (31–34). Rigorous studies do not treat passive data as a diagnostic fingerprint. Instead, they utilize these measures to investigate the relationship between real-world behavior and symptoms, functioning, environmental context, or treatment change (35). **Table 1** summarizes candidate passive sensing modalities and the digital phenotypes they generate. It details their construct relevance to experiential negative symptoms, key confounders, interpretation risks, and validation anchors linking them to clinical and ecological measures.

Mobility, activity, and device interaction patterns constitute the core behavioral measurement modalities. GPS-derived metrics, including time spent at home, distance traveled, number of locations visited, location entropy, and life-space range, indicate plausible indicators of behavioral activation, environmental exploration, and social opportunity (36–38). GPS-derived mobility indices show consistent but modest associations with negative symptoms, supporting their role as components of a multimodal phenotype rather than standalone endpoints (39) (**Table 1**). This pattern supports the utility of mobility data while arguing against reliance on single-modality endpoints. Complementing geolocation, wearables quantify steps, sedentary time, circadian regularity, sleep timing, sleep duration, and activity fragmentation. These signals are relevant to avolition, as experiential negative symptoms frequently involve diminished initiation and persistence of activity. Wearable studies confirm feasibility and adherence in schizophrenia, with activity signals correlating with negative symptoms yet requiring integration with clinical anchors (40) (**Table 1**). Collectively, these studies suggest that activity signals can enrich symptom measurement, yet they do not establish activity as a stand-alone digital endpoint. Phone interaction features, including screen use, application activity, charging patterns, mobility-phone coupling, and communication timing, also capture daily structure and behavioral inertia. However, interpreting these features is challenging due to the influence of age, income, culture, occupation, cognitive ability, and personal preference on device usage. Identical levels of phone use may indicate social engagement, avoidance, gaming, work, boredom, insomnia, or digital exclusion. These features become more informative when modeled relative to individual baseline and integrated with EMA, contextual data, and clinical information, as shown by evidence that mobility and communication metadata add explanatory value beyond EMA-rated interest and enjoyment for clinician-rated anhedonia and avolition (41).

Social sensing is central to the assessment of asociality, yet it presents significant methodological challenges. Clinically direct indicators include face-to-face interaction, time spent with others, visit patterns to social venues, communication frequency, response latency, and social network size. However, social data are also among the most sensitive. Although many platforms can collect communication metadata without content, even metadata alone can reveal intimate patterns. Recent work linking social network reductions to negative symptoms and loneliness in psychotic disorders strengthens the rationale for social sensing, while studies of intensive longitudinal social sensing demonstrate both promise and practical burden (42–44). Consequently, social signals should be treated as clinically valuable yet ethically high-risk data.

Speech and language occupy a related but distinct position. While alogia and expressive deficits are not the primary focus of this review, speech and language markers distinguish reduced social exposure from reduced expressive production. Automated speech analysis derived from clinical interviews, along with longitudinal speech or language markers, may quantify pauses, prosody, lexical diversity, semantic coherence, and symptom change (45, 46). The AMP SCZ initiative illustrates a strategy for collecting language, acoustic, and facial expression data to predict psychosis and other outcomes (47). Parola et al. demonstrated that voice markers of schizophrenia require cross-linguistic and cumulative validation rather than limited, single-language claims (48). Within a digital endpoint framework, speech should be interpreted as one modality within a multimodal model, rather than an isolated proxy for experiential symptoms.

Interpreting passive signals requires attention to environmental, clinical, and methodological context. James et al. demonstrated that ethnoracially incongruent environments predicted state increases in negative symptoms among individuals with schizophrenia (49). This finding positions context as a measurable component of the phenotype rather than a vague limitation. Treatment and medication data should also be integrated, as sedation, parkinsonism, and anticholinergic burden can alter passive signals in ways that mimic or mask negative-symptom change. A digital endpoint intended for a clinical trial must specify how concomitant medication, side effects, and intervention exposure are handled. The field must also distinguish between the absence of a signal and evidence of absence. An absence of outgoing calls does not imply a lack of social interaction; frequent messaging does not guarantee meaningful social engagement. These examples illustrate why passive phenotyping should be paired with carefully timed EMA to determine whether a social interaction was desired, effortful, or meaningful.

## 5 AI offers powerful tools but requires construct anchoring and rigorous validation

AI is frequently invoked in overly broad terms within the field of digital mental health. In this context, the value of AI should be assessed by its capacity to address measurement problems that simpler methods cannot resolve. Passive digital phenotyping yields data that are longitudinal, multimodal, irregularly sampled, highly sparse, and strongly person-specific. While a weekly average step count may offer utility, it fails to capture state transitions, modality interactions, contextual dependencies, or within-person deviations from baseline. AI and ML methods model these properties effectively when anchored to clinical constructs and validated against meaningful outcomes. Researchers have developed various modeling approaches to address distinct facets of this challenge, and the following subsections organize these approaches by function.

### 5.1 Traditional supervised learning and feature selection

Traditional supervised learning has been employed to identify digital phenotyping measures most relevant to negative symptoms. For instance, Narkhede et al. utilized ML to select digital measures associated with negative symptoms in psychotic disorders, explicitly framing their findings within the context of clinical trials (50). While such approaches are valuable for feature screening, they remain susceptible to overfitting, data leakage, unstable feature importance, and limited transportability, particularly when sample sizes are small. Consequently, their output should be regarded as hypothesis-generating unless externally validated and clinically interpretable.

### 5.2 Time-series analysis and personalized modeling

Beyond feature selection, mixed-effects, hierarchical, and idiographic models hold particular relevance for experiential negative symptoms. It is crucial to recognize that between-person differences and within-person changes are not interchangeable. Individuals who exhibit generally lower mobility may not be the same as those who demonstrate clinically meaningful deterioration relative to their own baseline. Within-person models can distinguish stable trait burden from deviations linked to context, medication, sleep disruption, stress, or social opportunity. EMA studies have demonstrated the significance of momentary and contextual modeling; passive sensing research should build upon these insights rather than reverting to crude cross-sectional classification.

At a higher level of complexity, time-series and deep learning models identify temporal states and transitions. These models can characterize the sequence of sleep disruption, reduced activity, diminished social contact, and symptom exacerbation. In principle, they also possess the capacity to learn early-warning features indicative of relapse or functional decline. Reviews on relapse prediction suggest that multimodal data and individual-level modeling frequently outperform single-modality and population-level approaches; however, the field continues to be dominated by internal validation and heterogeneous outcome definitions (20, 51). A similar risk pertains to negative symptoms: a model that predicts a future scale score within the same cohort does not yet constitute a transportable endpoint.

### 5.3 Multimodal fusion and self-supervised learning

Multimodal learning represents the most appropriate framework for avolition and asociality, as each symptom stems from multiple partial signals (52). Mobility, activity, sleep, social contact, speech, and context each convey limited information in isolation. When integrated, however, they distinguish reduced opportunity from reduced motivation, sedation from avolition, and social withdrawal from environmental exclusion. Multimodal biomedical AI has advanced rapidly, and general healthcare AI reviews emphasize the necessity of robust evaluation, calibration, and deployment-aware validation (52, 53). However, research in schizophrenia requires smaller yet more disciplined applications of this logic.

Emerging self-supervised learning approaches address the scarcity of labeled negative-symptom data by leveraging abundant unlabeled sensor streams to learn general representations adaptable to clinical outcomes (54, 55). However, transfer from general-population models to schizophrenia-spectrum disorders requires rigorous validation, as medication-induced motor abnormalities, hospitalization patterns, device access disparities, and altered social contexts can induce substantial domain shift. Future work must determine whether fine-tuning on small clinical samples suffices or whether disorder-specific pretraining is necessary.

### 5.4 Interpretability, calibration, and causal inference

Irrespective of model complexity, trustworthiness necessitates rigorous attention to interpretability, calibration, negative controls, and causal reasoning. Interpretability is indispensable. Clinical teams and patients must discern whether a model’s signal derives from reduced mobility, disrupted sleep, diminished social contact, missing data, or environmental shifts. Interpretable modeling should incorporate construct maps, modality-specific contributions, uncertainty estimates, and thresholds for model abstention. The goal is to address specific clinical questions rather than merely generate visualizations.

Calibration warrants greater emphasis than accuracy. A model designed to flag worsening avolition must estimate risk in a manner that informs clinical action; poor calibration precipitates alert fatigue or overlooked deterioration. Calibration should be evaluated globally and within subgroups (age, race, ethnicity, device type, illness stage); systematic overconfidence in underrepresented groups constitutes an equity concern.

Negative controls strengthen construct validity. If a mobility-derived endpoint measures avolition, it should be tested against constructs with weak theoretical associations or time windows lacking predictive validity. Such analyses can reveal confounding factors such as seasonality, device usage patterns, and socioeconomic status, and distinguish construct-specific signals from general illness markers. This distinction is critical in schizophrenia-spectrum disorders, where symptoms, cognition, medication effects, and social adversity are highly correlated.

Finally, patient-level counterfactual reasoning (a causal ML framework) remains underutilized. A clinically viable model should facilitate exploration of alternative explanations. Does reduced activity follow a medication change, or does it reflect low motivation? Does declining social contact stem from relocation or asociality? Context-aware AI must represent competing explanations to avoid premature pathologization of ordinary or structurally constrained behavior.

### 5.5 Summary and guiding principles

Digital psychiatry should adopt advanced AI methods with restraint. In small schizophrenia cohorts, fine-tuning powerful representations risks leaving clinical targets poorly defined. A robust approach entails pairing modern representation learning with conservative validation strategies, including prespecified endpoints, external cohorts, modality ablations, and uncertainty quantification. **Table 2** summarizes method families, clinical questions, validation requirements, and failure modes.

**Table 2:** AI/ML methods, clinical questions, validation needs, and failure modes.

Method family	Best-suited clinical question	Validation requirements	Common failure modes
Supervised feature-based models	Which passive features are associated with avolition or asociality?	External validation, stability of selected features, construct anchoring, calibration	Overfitting, leakage, unstable feature importance, small-sample bias (50, 51, 58)
Mixed-effects and hierarchical models	Which effects are between-person and which are within-person?	Correct temporal alignment, repeated assessments, person-level baselines	Conflating trait differences with state change, ignoring contextual clustering (24–27, 51)
Time-series and sequence models	Can trajectories predict worsening, functional decline, or treatment response?	Prospective validation, time-window justification, missingness modeling, calibration	Black-box predictions, insufficient data, unreported uncertainty, poor transportability (51, 81, 82)
Multimodal fusion	Do mobility, activity, social, speech, and context signals add complementary value?	Modality-ablation tests, missing-modality robustness, interpretable fusion	One dominant modality, hidden confounding, performance collapse when a sensor fails (41, 52, 53, 59)
Self-supervised representation learning	Can unlabeled sensor streams support robust phenotypes with limited labels?	Domain-shift testing, fine-tuning validation, subgroup evaluation	General-population representations that fail in SSD, opaque embeddings, biased pretraining data (54, 55)
Personalized anomaly detection	Is a patient deviating from their own baseline in a clinically meaningful way?	Stable baseline period, false-alarm evaluation, clinical actionability	Mistaking ordinary life changes for symptom worsening, alert fatigue, weak interpretability (79, 81, 82)
Context-aware models	Is low activity or low social contact better explained by symptoms, environment, or opportunity?	Geocoded context, ecological covariates, multilevel modeling, privacy safeguards	Pathologizing poverty, social exclusion, cultural norms, or unsafe environments (24, 25, 49, 83, 85)

## 6 Digital markers must meet rigorous validation standards to qualify as clinical endpoints

The distinction between a digital marker and a digital endpoint is critical. A digital marker may correlate with symptoms, whereas a digital endpoint must be fit for a defined clinical purpose. In clinical trials, a digital endpoint must be technically reliable, analytically valid, clinically interpretable, sensitive to change, and meaningful within the specified context of use. Goldsack et al. formalized verification, analytical validation, and clinical validation, often summarized as V3, as the foundation for fit-for-purpose biometric monitoring technologies (56). Similarly, Kruizinga et al. emphasized the value-based, structured validation of digital endpoints, while TRIPOD+AI specifies reporting expectations for prediction models that use regression or machine-learning methods (57, 58). These frameworks should be central to digital phenotyping of negative symptoms.

Verification assesses whether devices and data pipelines accurately measure raw signals, including sensor quality, timestamp integrity, non-wear detection, and platform-specific constraints (59, 60). Failures at this foundational level render downstream AI analyses meaningless. Analytical validation examines whether derived features and model outputs are accurate and robust across preprocessing, missing data handling, feature definitions, calibration, and device variability. Missingness is both a data quality issue and a potential clinical signal; in schizophrenia, device disengagement may indicate paranoia, hospitalization, or symptom worsening rather than solely technical failure (61). Resolution mismatch poses a critical challenge: passive sensors generate minute-level data, while negative-symptom scales are administered every few weeks (62). For experiential negative symptoms, the relevant temporal unit may be a morning activation pattern, a day-level social opportunity, or a month-level functional trajectory. **Figure 2** illustrates how inappropriate aggregation can obscure avolition and asociality signals (**Figure 2**). Clinical validation determines whether a digital measure reflects the intended construct and improves decision-making within the specific context of use. For avolition and asociality, validation must extend beyond correlation with total negative-symptom scores to include the motivation and pleasure dimension, EMA-derived motivation and social context, clinician-rated change, functional outcomes, patient-valued activities, and treatment response (63). The field requires convergent and discriminant validation; digital endpoints should demonstrate stronger relationships with avolition and asociality than with unrelated constructs, while acknowledging overlap with depression, sedation, and extrapyramidal symptoms. Clinical validation should incorporate failure analysis, reporting instances where digital endpoints diverge from clinical ratings (64). For example, a patient may report improved motivation while mobility remains low due to physical illness, or exhibit increased activity driven by agitation rather than goal-directed engagement.

**Figure 2:**
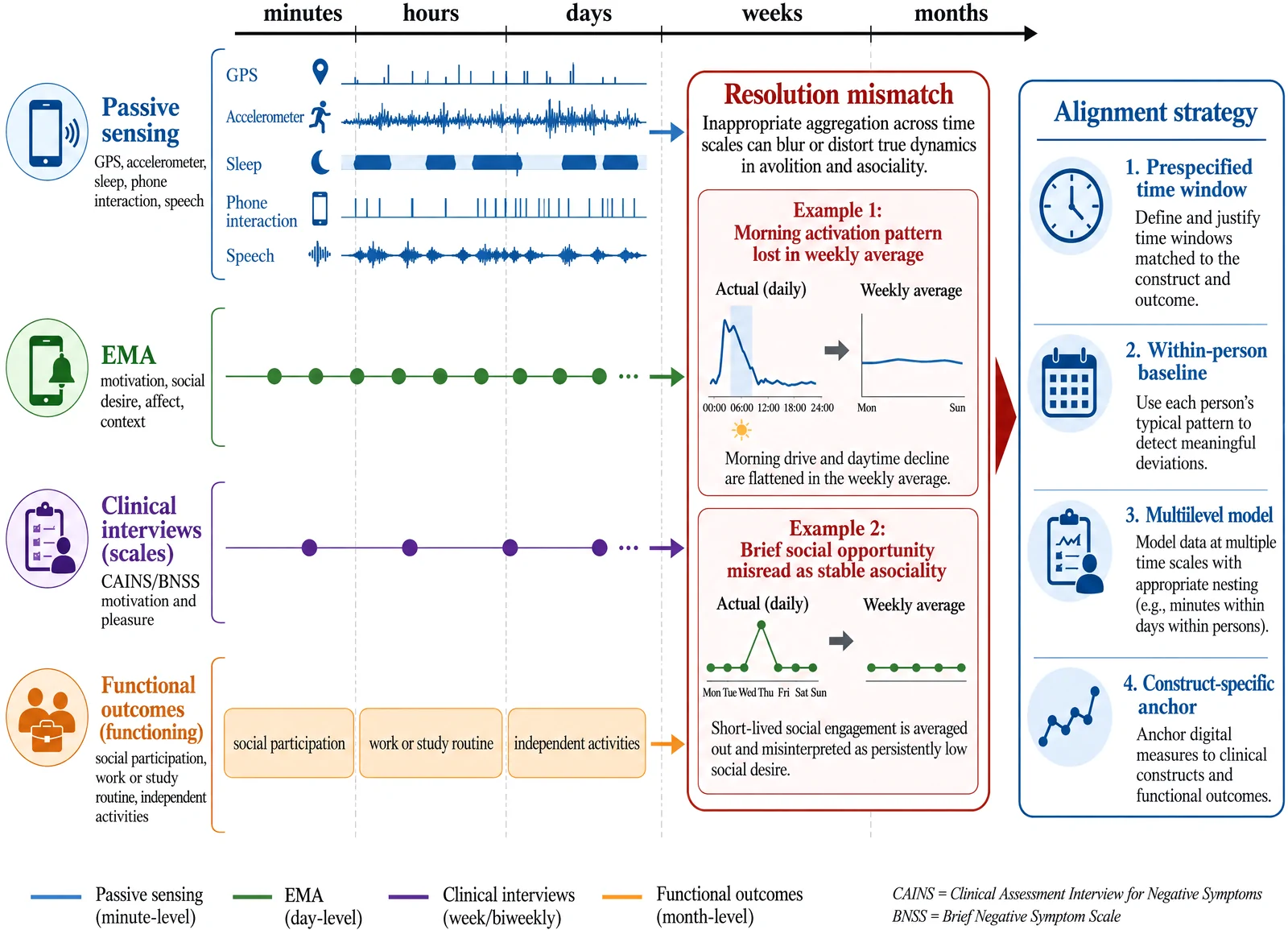
Resolution mismatch in negative-symptom digital phenotyping. This figure illustrates minute-level passive sensor data, day-level EMA and social context, week-level negative-symptom interviews, and month-level functional outcomes, providing examples of how aggregation can obscure or distort signals related to avolition and asociality. For instance, Meyer et al. (82) demonstrated that sleep disturbance and psychopathology in psychosis operate through day-to-day temporal dynamics with specific directional lags; aggregating such data into weekly or monthly summaries would mask these clinically informative transitions and collapse distinct temporal sequences into a single static estimate. Source: Created in Microsoft PowerPoint. Icons and illustrations were adapted from open-license resources, with modifications limited to size, color, layout, and style consistency.

Patient meaningfulness constitutes the ultimate determinant of utility. A digital endpoint is not beneficial merely because it responds to treatment; it must reflect changes that are meaningful to the patient. Examples include leaving home to attend rehabilitation, sustaining work or study routines, engaging in chosen social interactions, participating in family life, shopping independently, or maintaining a sleep-wake rhythm that supports daily functioning. Methods for determining patient meaningfulness include patient-nominated goals, qualitative interviews, or patient-reported outcome measures that assess the perceived impact of digital changes on daily life (65). Schooler et al. emphasized the necessity of defining therapeutic benefit for negative symptoms (66), while Marder and Kirkpatrick highlighted the measurement challenges inherent in negative-symptom trials (67). Digital endpoints can advance this agenda only if they are linked to outcomes that individuals with schizophrenia-spectrum disorders recognize as valuable.

The FDA guidance on digital health technologies emphasizes context of use, data integrity, privacy, and reliability (68). For negative-symptom trials, a narrow initial scope is advisable. For example, an exploratory endpoint for daytime activity structure in stable outpatients with prominent avolition is more tractable than a general endpoint spanning all settings and devices. A comprehensive digital endpoint package should comprise four dossiers: technical (sensors, preprocessing, quality control), analytical (feature definitions, calibration, missing data handling), clinical (construct validity, sensitivity to change, patient meaningfulness), and implementation (display, interpretation, clinical action). Most current studies address only select components of the first three dossiers. The endpoint should also define the magnitude of change that constitutes a meaningful difference, potentially anchored to patient-nominated goals and clinician-rated improvement.

## 7 The evidence base demonstrates feasibility and associations but requires targeted endpoint development

The evidence reviewed here converges on a measured position. Passive and active digital data collection is feasible in schizophrenia-spectrum disorders, yet feasibility alone does not establish endpoint validity for experiential negative symptoms. Associations between digital measures and negative symptoms are consistent but modest, and recent work incorporating environmental context has begun to reframe the field beyond individual-deficit models. The gap between data abundance and endpoint readiness becomes increasingly consequential.

Three conclusions emerge from the evidence reviewed above. First, feasibility is established more firmly than endpoint validity. Patients can provide passive smartphone, wearable, GPS, EMA, and speech data, though adherence varies and missingness remains significant. Second, GPS and actigraphy studies support links between restricted activity and negative symptoms, but effect sizes are modest because passive signals are multiply determined. Without clinical anchors and contextual data, single-modality correlations cannot distinguish avolition from depression, sedation, or environmental constraints. Digital endpoints for avolition and asociality must therefore integrate multiple modalities and contextual information. Third, environmental context predicts state fluctuations in negative symptoms beyond individual factors, and digital phenotyping of reduced positivity offset illuminates reward processes linked to anhedonia (24, 25, 69). These findings shift the field toward context-sensitive models of experiential negative symptoms.

Digital phenotyping has become increasingly integrated into treatment research on negative symptoms. Harvey et al. embedded EMA and actigraphy in a 52-week open-label trial of xanomeline and trospium chloride, observing changes in sedentary behavior, physical activity, and social functioning (70, 71). However, the open-label design precludes causal attribution; whether behavioral shifts reflect pharmacological benefit, practice effects, or non-specific engagement remains uncertain pending controlled replication.

Digital therapeutics targeting negative symptoms are advancing from feasibility to larger trials, including CT-155/BI 3972080 and app-based or augmented-reality interventions (72–76). These interventions can use passive data to tailor content and timing; however, tailoring effectiveness depends on the validity of the underlying digital phenotype. Digital therapeutics and digital phenotyping should therefore be developed in tandem. Looking ahead, social and environmental phenotyping and speech markers represent the most promising methodological advances. Their value depends on integration within the multimodal framework outlined above rather than deployment as standalone measures.

In summary, passive digital phenotyping has advanced beyond proof of concept and can capture clinically relevant behavior, with AI methods aiding the modeling of complex trajectories. However, the field has not yet produced qualified digital endpoints for avolition or asociality. The near-term priority is targeted endpoint development in defined contexts of use, paired with construct-anchored validation.

## 8 Clinical translation: trials, monitoring, and JITAIs

The most realistic near-term role for passive digital phenotyping is augmenting, not replacing, clinician-rated scales. In early clinical development, passive data serves as exploratory biomarkers to identify behavioral patterns associated with avolition and asociality. In later-phase trials, rigorously validated digital measures may function as secondary endpoints that complement the CAINS, BNSS, EMA, and functional assessments. Qualified digital endpoints may eventually support enrichment, monitoring, or treatment-response assessment within specified contexts of use.

Within clinical trials, passive digital phenotyping strengthens endpoint design, sample selection, and mechanism testing. Researchers should begin trials with a clear endpoint statement. For example, a trial might evaluate whether a treatment increases time spent in non-home locations during personally meaningful daytime windows among participants with high avolition, while concurrently improving CAINS motivation and pleasure, EMA motivation, and patient-nominated activity goals. This approach is more robust than testing whether the treatment increases raw step counts in all participants, as it specifies the construct, population, time window, clinical anchor, and patient relevance. Beyond endpoint design, trial enrichment offers a particularly valuable application. Many negative-symptom trials are hindered by heterogeneity; passive digital phenotyping could help identify participants with stable experiential negative-symptom burden, restricted activity space, low social engagement, and adequate device adherence prior to randomization. For example, a trial might require participants to demonstrate at least two weeks of stable low mobility combined with CAINS motivation and pleasure scores above a defined threshold. Such enrichment would not replace clinical eligibility criteria, but reduces noise and enhances the likelihood of detecting treatment effects. Furthermore, passive data can facilitate mechanism testing. If a psychosocial intervention is designed to improve goal pursuit, passive measures can determine whether participants exhibit increased initiation, activity diversity, and routine regularity before broader functional gains emerge. Similarly, if a digital therapeutic targets social motivation, passive and EMA measures can assess whether social desire, utilization of social opportunities, and actual interactions change concurrently. This mechanism-oriented approach is more informative than treating passive data as a generic outcome stream.

Beyond the trial context, passive digital phenotyping can facilitate clinical monitoring, adaptive intervention delivery, and digital therapeutics (77, 78). Routine monitoring frameworks may be implemented prior to regulatory endpoint qualification. A passive monitoring system alerts clinicians to sustained reductions in mobility, social rhythms, or activity levels relative to a patient’s baseline. The appropriate clinical response should involve inquiry rather than automatic interpretation. Clinicians might investigate potential factors such as sedation, depression, paranoia, physical illness, transportation barriers, social stress, or environmental changes. In this model, AI generates a structured prompt for shared decision-making; it does not diagnose avolition. Extending monitoring further, JITAIs provide a longer-term translational pathway. JITAIs utilize decision points, tailoring variables, intervention options, and decision rules to deliver support when it is most likely to be effective (79). For experiential negative symptoms, interventions trigger when several days of reduced activity combine with EMA-reported low motivation, social contact decreases alongside preserved desire for connection, or disrupted sleep precedes declining daytime engagement. Potential responses include micro-goal setting, behavioral activation, social approach tasks, sleep regularity support, digital therapeutic modules, or low-intensity clinician outreach. Human oversight is essential, as false alarms may burden patients, and certain digital patterns may reflect rational adaptations to unsafe or stressful contexts. Digital therapeutics render this linkage particularly critical. Fulford et al. described prescription digital therapeutics as an emerging option for negative symptoms, and feasibility and trial-protocol studies are now available for experiential negative symptoms in schizophrenia (80). These interventions require both passive and active measures to evaluate engagement, mechanisms, and treatment response. The measurement layer should be designed concurrently with the intervention, rather than appended as an afterthought.

Translating these applications into clinical practice requires a phased implementation strategy with careful consideration of clinical workload. In the initial stage, passive phenotyping serves as a descriptive tool during consultations, analogous to a sleep diary or activity log. Clinicians and patients can collaboratively examine behavioral patterns to determine whether they reflect symptoms, side effects, therapeutic goals, or contextual factors. In the second stage, validated summaries should be integrated into measurement-based care, utilizing thresholds that prompt clinical assessment rather than triggering automatic intervention. In the third stage, individualized models can support treatment planning, relapse prevention, and delivery of JITAIs. Premature advancement to this third stage risks eroding trust and increasing the likelihood of behavioral misclassification. Throughout each phase, implementation must account for clinical workload constraints. Systems generating daily risk scores without defined response pathways may exacerbate administrative burden and liability. A more effective model involves tiered feedback: low-level summaries such as weekly activity reports to facilitate patient self-reflection; intermediate alerts for sustained deviations from baseline that prompt clinical review; and higher-risk alerts based on individually calibrated, multi-domain warning signals, such as concurrent deterioration in mobility, sleep, and social rhythms. Earlier pilot work found that behavioral anomalies were 71% more frequent during the two weeks preceding relapse (81), while digital sleep sampling showed that poorer sleep quality and shorter sleep duration predicted worsening psychosis symptoms over subsequent 1–8 and 1–12 day windows, respectively (82). These findings support the use of individually calibrated warning signals, but they do not establish universal clinical alert cut-points. For high-risk alerts, the system should define deviation criteria calibrated to each patient’s own behavioral baseline and recommend assessment questions such as whether anything has changed in the living situation or whether the patient is experiencing increased paranoia or depression. This approach maintains the clinician within the decision-making loop, preventing the model from functioning as an unexamined authority.

## 9 Ethical digital phenotyping requires privacy protection, bias mitigation, and equitable implementation

Passive digital phenotyping in psychosis raises heightened privacy and trust concerns, as GPS, social metadata, speech, and phone interaction data can reveal residence, social contacts, sleep patterns, and periods of disengagement. Ethical digital phenotyping requires data minimization, transparent and renewable consent, modality-specific opt-in/opt-out, encryption, role-based access, and clear restrictions on secondary use (83). These safeguards directly influence adherence, representativeness, and clinical legitimacy. Consent should be conceptualized as an ongoing relationship rather than a singular event, as the implications of data collection may shift with symptom fluctuations over weeks or months. An individual comfortable sharing step counts might be unwilling to share GPS data, social metadata, or voice recordings. Consent systems should therefore facilitate modality-specific choices and allow for pauses in data collection. These systems must also clarify protocols for when data suggest clinical deterioration, including whether clinicians will be notified and whether results will be returned to participants (84). Data minimization is particularly critical for social and speech data, as many clinical inquiries can be addressed using metadata or derived features rather than raw content. These methodological choices must be justified by the specific clinical question and validation plan. If the research question concerns social contact frequency, metadata suffice; if it concerns desire for social contact despite available opportunities, EMA data on social context and motivation becomes necessary, but raw message content remains unnecessary. Collecting data in excess of what is required constitutes ethical overreach, not scientific rigor.

Beyond individual-level privacy, the field must address systematic biases that can distort digital phenotypes and undermine equity. Algorithmic bias constitutes a central scientific risk. Digital phenotypes are shaped by variables such as device ownership, data plans, employment, housing, disability, race, ethnicity, neighborhood resources, language, and culture. A model trained in a well-resourced urban clinic does not generalize to a rural setting or a population with limited data access. More critically, a model can pathologize structural disadvantage by misinterpreting reduced mobility or social contact as negative symptoms. For example, a patient living in a high-crime neighborhood exhibits low mobility due to safety concerns, not avolition. A patient from a collectivist culture exhibits low individual social contact because social interaction occurs primarily within extended family households, not due to asociality. A model that fails to account for these contextual factors systematically overestimates negative symptom severity in marginalized groups. To mitigate this risk, studies should recruit diverse samples across socioeconomic, racial, ethnic, and geographic strata; collect contextual data on neighborhood safety, social opportunity, and cultural norms; evaluate model performance within subgroups; report calibration and fairness metrics; and involve patients from underrepresented groups in endpoint design and interpretation. Mulinari argued that digital biomarker definitions in psychiatry should align with construct frameworks such as the National Institute of Mental Health Research Domain Criteria; however, construct alignment must also incorporate social and environmental context (85). **Figure 3** illustrates this problem, demonstrating how identical signals of low activity or social contact may stem from negative symptoms, medication side effects, depression, physical illness, cognitive impairment, neighborhood resources, social opportunity, cultural norms, ethnoracial incongruence, or device-related missingness (**Figure 3**).

**Figure 3:**
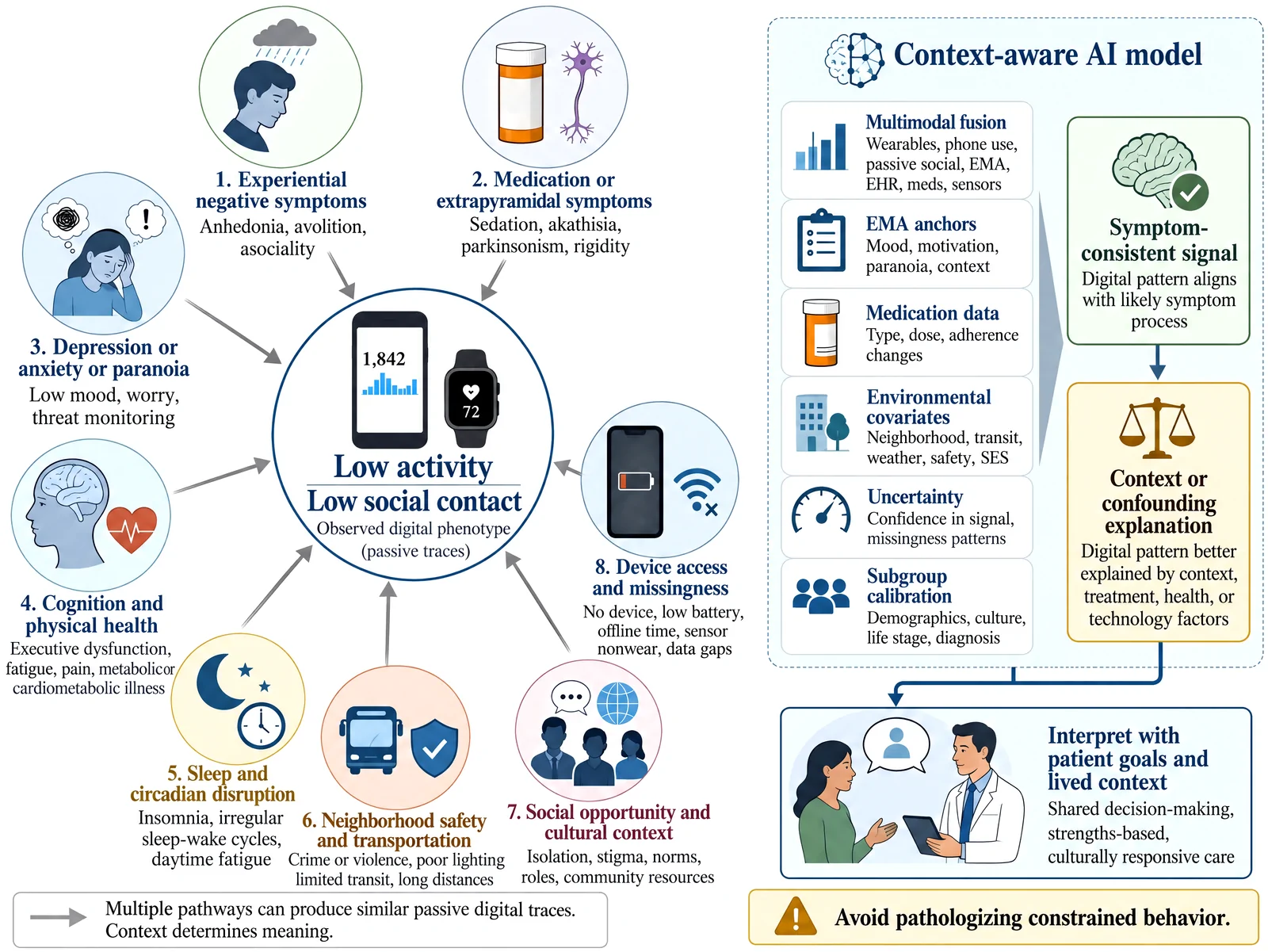
Context-aware model of avolition and asociality. This figure illustrates how identical signals of reduced activity or diminished social contact may stem from negative symptoms, medication effects, depression, physical illness, cognitive impairment, neighborhood resources, social opportunities, cultural norms, ethnoracial incongruence, and device-related missingness. Source: Created in Microsoft PowerPoint. Icons and illustrations were adapted from open-license resources, with modifications limited to size, color, layout, and style consistency.

Missingness compounds this risk and should be treated as both a design challenge and a potential clinical variable. Informative missingness may result from paranoia, low motivation, hospitalization, social instability, poverty, or deliberate withdrawal from monitoring. Models that impute missing data without understanding these mechanisms yield biased and overconfident outputs. Reporting should detail missingness by modality, time, participant subgroup, and clinical state. Equity should therefore be measured rather than merely discussed. Studies must report recruitment and retention metrics by demographic and socioeconomic variables, device ownership, data-plan stability, and digital literacy. Models should be evaluated across diverse subgroups and environments. If performance discrepancies exist, investigators should determine whether they reflect biased sampling, device inequity, feature definitions, or clinical heterogeneity.

Even technically robust and equitable models will fail to improve care if they cannot be integrated into clinical workflows and trusted by patients and clinicians. Clinicians require concise, interpretable summaries rather than streams of raw data; these summaries should identify significant changes, indicate model confidence, suggest alternative explanations for observed patterns, and recommend reasonable actions. Patients need to understand what data are collected and how they are interpreted, as well as whether they possess the rights to view, correct, pause, or delete their information (86). A digital endpoint that is statistically elegant but unusable within clinical workflows will not improve care. Trust also depends on the management of errors. False positives may precipitate unnecessary anxiety, stigma, or intrusive outreach, whereas false negatives may foster false reassurance. Patients fear that low activity levels will be interpreted as nonadherence or lack of effort, or that data might be disclosed to family members, insurers, employers, or legal authorities. These concerns are not irrational, as psychiatric data carry significant social consequences. Consequently, governance frameworks must explicitly define who can access digital phenotyping outputs, the duration of data storage, protocols for handling incidental findings, and the mechanisms by which patients can challenge interpretations.

## 10 A construct-to-endpoint AI pipeline

Translating digital phenotyping into qualified endpoints requires sequential attention to construct definition, signal justification, temporal modeling, multimodal integration, and cumulative validation. Each stage carries distinct risks of misalignment between measured parameters and claimed constructs. The pipeline begins with explicit specification of the target construct. Studies must state whether the target is avolition, asociality, anticipatory anhedonia, or another experiential dimension, as these overlapping constructs require different passive measures and clinical anchors. Signal selection should follow, guided by plausible construct relationships rather than ease of collection. Cohen et al. argued that biobehavioral technologies advance negative-symptom research precisely because they capture behavior at levels that rating scales cannot reach (87). This promise, however, depends on rigorous validation of each signal against its target construct (88).

Temporal alignment requires defining the time scale at which the model operates (momentary, daily, or monthly), and sensitivity analyses should assess robustness across aggregation windows. Multimodal modeling integrates sensor streams, EMA, clinical ratings, medication, environmental data, and patient-reported goals. At the same time, such modeling risks overfitting and producing clinically implausible inferences. The model should therefore incorporate prespecified modality groups, feature-ablation analyses, calibration plots, and subgroup performance metrics. When a model posits that reduced activity indicates worsening avolition, it must demonstrate that this association persists after adjusting for sleep, depressive affect, medication changes, and environmental opportunity.

Endpoint interpretation should translate model outputs into standard clinical language. For example, a report might state that relative to baseline, the patient spent fewer daytime hours away from home and reported lower motivation on EMA for six consecutive days. Such a description conveys more clinical meaning than a latent engagement score. Evidence accumulation proceeds from feasibility and face validity through reliability, convergent and discriminant validity, sensitivity to change, and transportability. This process culminates in trials testing whether the endpoint responds to interventions targeting avolition or asociality.

This pipeline also delineates practices that AI should avoid. It should not transform passive sensing into automated clinical judgment, supplant patient dialogue, obscure uncertainty, or make normative assumptions regarding movement, sociability, or phone use. Artificial intelligence demonstrates the greatest utility when it renders dense behavioral data intelligible and testable within a clinical framework.

## 11 Future directions and limitations

### 11.1 Future directions

The field requires progress on multiple fronts, but three priorities stand out as most urgent. Studies must pair passive sensing with CAINS or BNSS, EMA, functional outcomes, and patient-nominated goals to establish convergent and discriminant validity. Models require testing across devices, centers, cultures, and seasons to establish transportability. Individuals with schizophrenia-spectrum disorders must be involved in defining meaningful endpoints, acceptable sensing modalities, and actionable feedback. To advance these priorities, researchers should develop a construct-to-signal dictionary specifying which passive features correspond to each experiential negative-symptom subdomain, listing plausible confounders, and recommending validation anchors. Such a resource would discourage the current practice of indiscriminately labeling mobility features as negative-symptom markers. Validation should become multilayered by default, reporting convergent, discriminant, predictive, and treatment-sensitivity evidence rather than relying on baseline correlations with total negative-symptom scores. Models should prioritize within-person change, as a patient-specific decline from a stable baseline may hold greater clinical meaning than comparison with population averages. External validation should become routine, with model reports adhering to TRIPOD+AI guidelines and intervention studies employing CONSORT-AI and SPIRIT-AI standards (58, 89–91).

Emerging methods must be evaluated with clinical discipline. Sensor foundation models and self-supervised learning require cautious evaluation, as large-scale pretraining may import biases from healthier or more digitally connected populations. Digital endpoints should be embedded in trials and JITAIs that test actionability by identifying who benefits, when treatment changes daily engagement, or when timely support prevents decline. Micro-randomized trials are particularly suited to testing intervention timing in JITAIs. Equally important, patient partnership must be prioritized earlier in the development process, with patients actively participating in defining meaningful behavioral changes, acceptable sensing modalities, and appropriate feedback. Without patient-centered design, passive digital phenotyping risks becoming surveillance rather than a tool for recovery.

### 11.2 Limitations

We did not conduct formal quality appraisal of included studies using standardized risk-of-bias tools. Instead, evidence was synthesized based on construct-to-endpoint logic and methodological rigor as judged by the authors. This approach was chosen because the field is still at an early stage of development and because our focus was on translational validity rather than pooled effect estimation. The evidence base is rapidly evolving. New passive sensing studies, digital endpoints, AI models, and clinical trials are emerging frequently, and this review draws on literature available through May 2026. Findings and recommendations may require updating as the field matures, particularly regarding validation frameworks, regulatory guidance, and clinical implementation. We drew primarily on English-language publications, which introduces language and publication bias. Studies published in other languages or in grey literature may report different findings, implementation challenges, or cultural considerations that we have not captured. Finally, several cited studies are small feasibility or pilot investigations whose generalizability remains unclear. Many report associations between passive measures and negative symptoms in relatively homogeneous samples, often from single centers or specific geographic regions. External validation across diverse populations, settings, devices, and illness stages is limited. Consequently, the extent to which current findings will replicate in broader clinical populations, real-world care settings, and regulatory contexts is uncertain. Larger, multicenter, and more diverse studies are needed to establish the robustness and transportability of digital phenotyping measures for avolition and asociality.

## 12 Conclusion

AI-enabled passive digital phenotyping can deepen measurement of experiential negative symptoms, but only if the field resists simple sensor-to-symptom equations. Avolition and asociality are not step counts, GPS entropy, call frequency, or speech pauses. They are clinical constructs expressed through daily behavior under specific social, environmental, and biological conditions. The next step is to validate AI-derived, multimodal, context-aware, and interpretable digital markers as fit-for-purpose endpoints anchored to CAINS or BNSS, EMA, functioning, treatment response, and patient-valued change (56, 57, 63, 64, 66, 68). If this standard is met, passive digital phenotyping will strengthen negative-symptom trials and enable personalized monitoring and JITAIs support.
